# Supplementary material for: Performance of the UNICEF/UN Washington Group tool for identifying functional difficulty in rural Zimbabwean children
Source: PLoS One. 2022 Sep 16;17(9):e0274664. doi: 10.1371/journal.pone.0274664 (PMC9480986; doi:10.1371/journal.pone.0274664)
Supplement: S1 Methods — (DOCX) [file pone.0274664.s001.docx]

## **Supplementary methods**

Nurses underwent three weeks of residential training in assessment of ECD and had further refresher training and standardisation every six months. 6-monthly standardisation utilised non-SHINE trial children and involved nurses undertaking ECD assessment whilst observed and double-marked by a gold-standard assessor. A percentage agreement of >85% was required to pass. Intra-class correlations were also conducted at each refresher by asking nurses to measure one child twice (in the morning and the afternoon). MDAT intra-class correlation was 0.88 (95% CI= 0.82, 0.94). In-field validity checks were also conducted. Research nurses were subjected to monthly research visits where supportive supervision of ECD assessments were undertaken providing feedback to nurses. In addition to this, 5% of field assessments were video recorded, with footage reviewed and double-marked by a psychologist with expertise in all SHINE trial tests and a paediatrician with training in neurodevelopment and Shona language proficiency. Percentage agreement was 93% for MDAT fine motor and 90% for MDAT language.
