# Supplementary material for: Performance of the UNICEF/UN Washington Group tool for identifying functional difficulty in rural Zimbabwean children
Source: PLoS One. 2022 Sep 16;17(9):e0274664. doi: 10.1371/journal.pone.0274664 (PMC9480986; doi:10.1371/journal.pone.0274664)
Supplement: S1 Table — (DOCX) [file pone.0274664.s002.docx]

**Supplementary Table 1. Baseline characteristics of mothers and children enrolled into the disability compared to mothers and children not enrolled into disability study.**

| **Baseline Characteristic** | Enrolled into disability study | Not enrolled into disability study | P value |
| --- | --- | --- | --- |
| Women assessed, N | 1902 | 2473 |  |
| Children assessed, N | 1920 | 2507 |  |
| Women completing baseline visit, N | 1814 | 2249 |  |
| **Household characteristics** |  |  |  |
| Size, median (IQR) [n] | 5 (3,6) [1801] | 4 (3,6) [2389 |  |
| Wealth quintile, percent [n] |  |  |  |
| Lowest | 18.9 [341] | 19.7 [439] | 0.300 |
| Second | 19.4 [350] | 20.4 [453] |  |
| Middle | 21.0 [379] | 19.8 [440] |  |
| Fourth | 21.2 [382] | 19.3 [430] |  |
| Highest | 19.5 [351] | 20.8 [462] |  |
| ***Electricity*** |  |  |  |
| Power grid, percent [n] | 3.1 [55] | 2.6 [57] | 0.320 |
| Other power, percent [n]: |  |  |  |
| Generator | 3.1 [56] | 3.3 [73] | 0.661 |
| Solar | 67.1 [1210] | 65.4 [1455] |  |
| No electricity | 28.1 [506] | 29.7 [660] |  |
| ***Sanitation*** |  |  |  |
| Household members who openly defecate (all), percent [n/N] | 48.7 [4035/8280] | 49.2 [5240/10642] | 0.070 |
| Household members who openly defecate (by age group), percent [n/N] |  |  |  |
| 0 to <3 years | 7.8 [300/3856] | 7.8 [413/4991] | 0.149 |
| 3 to <6 years | 12.0 [464/3856] | 12.0 [542/4991] |  |
| 6 to <18 years | 26.4[1016/3856] | 26.4[1220/4991] |  |
| 18 to <70 years | 44.8[1727/3856] | 44.8[2304/4991] |  |
| >70 years | 9.1 [349/3856] | 9.1 [512/4991] |  |
| Any latrine at household, percent [n] | 36.8 [648] | 36.5 [806] | 0.821 |
| Improved latrine at household, percent [n] | 32.8 [577] | 31.3 [691] | 0.299 |
| Improved latrine with well-trodden path, percent [n] | 29.0 [510] | 27.3 [603] | 0.215 |
| Improved latrine with well-trodden path and not shared, percent [n] | 26.4 [449] | 24.6 [525] | 0.200 |
| ***Water*** |  |  |  |
| Main source of household drinking water is improved, percent [n] | 62.4 [1102] | 63.3 [1404] | 0.656 |
| Treat drinking water to make it safer, percent [n] | 14.3 [248] | 11.4 [249] | 0.012 |
| One-way walk time to fetch water (min), median (IQR) [n] | 10 (5,15) [1761] | 10 (5,20) [2214] | 0.012 |
| Per capita water volume collected past 24 h (l), mean (SD) [n] | 41.8 (38.2) [1548] | 43.4 (53.3) [1900] | 0.164 |
| ***Hygiene*** |  |  |  |
| Handwashing station at household, percent [n] | 9.7 [163] | 8.6 [181] | 0.320 |
| Handwashing station with water, percent [n] | 2.9 [48] | 3.2 [67] | 0.525 |
| Handwashing station with water and rubbing agent, percent [n] | 0.9 [15] | 0.5 [11] | 0.110 |
| Improved floor, percent [n] | 53.7 [956] | 54.7 [997] | 0.549 |
| own chickens, percent [n] | 80.7 [1459] | 79.0 [1761] | 0.151 |
| Livestock observed inside the house, percent [n] | 38.9 [726] | 35.6 [851] | 0.032 |
| Faeces observed in the yard, percent [n] | 32.6 [605] | 30.0 [715] | 0.076 |
| ***Diet quality and food security*** |  |  |  |
| Household meets minimum dietary diversity score, percent [n] | 40.9 [644] | 38.7 [756] | 0.197 |
| Coping Strategies Index, median (IQR) [n] | 1 (0,8) [1763] | 1 (0,7) [2167] | 0.590 |
| **Maternal characteristics** |  |  |  |
| Age (y), mean (SD) [n] | 27.2 (6.8) [1705] | 25.6 (6.4) [2176] | <0.001 |
| Height (cm), mean (SD) [n] | 159.8 (9.5) [1863] | 160.0 (7.7) [2388] | 0.640 |
| MUAC (cm), mean (SD) [n] | 26.5 (3.1) [1881] | 26.3 (3.0) [2408] | 0.216 |
| Completed schooling (y), mean (SD) [n] | 9.5 (1.8) [1788] | 9.5 (1.8) [2347] | 0.816 |
| Parity, median (IQR) [n] | 2 (1,3) [1406] | 2.0 (1,3) [1641] | <0.001 |
| Married, percent [n] | 95.6 [1699] | 94.8 [2206] | 0.210 |
| Employed, percent [n] | 9.5 [170] | 8.0 [179] | 0.131 |
| Religion, percent [n] |  |  |  |
| Apostolic | 49.8 [890] | 44.6 [1046] | <0.001 |
| Other Christian (Pentecostal, Catholic, other Christian) | 43.3 [774] | 46.0 [1080] |  |
| Other religions (Muslim and other) | 6.9 [124] | 9.5 [222] |  |
| **HIV status, percent [n]** |  |  |  |
| Positive | 16.4 [311] | 14.0 [346] | <0.001 |
| Negative | 83.7 [1591] | 83.4 [2056] |  |
| Unknown | 0.0 [0] | 2.9 [71] |  |
| Maternal capabilities |  |  |  |
| Gender norms and attitudes, median (IQR) [n] | 1.8 (1.5;3.0) [1792] | 1.7 (1.5;3.0) [2207] | 0.001 |
| Perceived social support, median (IQR) [n] | 3.7 (3.3;4.0) [1766] | 3.6 (3.2;4.0) [2154] | 0.001 |
| Perceived physical health, median (IQR) [n] | 3.6 (2.8;4.2) [1574] | 3.6 (2.7;4.2) [1917] | 0.215 |
| Mothering self-efficacy, median (IQR) [n] | 4.0 (3.8;4.2) [1774] | 4.0 (3.7;4.2) [2159] | 0.007 |
| Perceived time stress, median (IQR) [n] | 2.6 (2.0;3.2) [1767] | 2.6 (2.0;3.2) [2166] | 0.610 |
| Decision-making autonomy, median (IQR) [n] | 5.0 (4.0,5.0) [1609] | 5.0 (3.0,5.0) [2037] | 0.018 |
| **Infant characteristics** |  |  |  |
| Female, percent [n] | 50.0 [959] | 50.1 [1256] | 0.928 |
| Birth weight (kg), mean (SD) [n] | 3.1 (0.5) [1830] | 3.1 (0.5) [2227] | 0.408 |
| Birth weight <2500g, percent [n] | 8.9 [162] | 8.8 [195] | 0.916 |
| Institutional delivery, percent [n] | 89.3 [1624] | 88.1 [2005] | 0.231 |
| Vaginal delivery, percent [n] | 92.7 [1729] | 92.4 [2120] | 0.305 |
