# Supplementary material for: Performance of the UNICEF/UN Washington Group tool for identifying functional difficulty in rural Zimbabwean children
Source: PLoS One. 2022 Sep 16;17(9):e0274664. doi: 10.1371/journal.pone.0274664 (PMC9480986; doi:10.1371/journal.pone.0274664)
Supplement: S2 Table — (DOCX) [file pone.0274664.s003.docx]

**Supplementary Table 2. Table reporting frequency of responses for each question of the Washington Group/UNICEF Module on Child Functioning (2015 version) from HIV unexposed infants.**

|  | **Question from WGCFM, version for 2-4-year-olds**  **n (%)** | | | | | | | | | |
| --- | --- | --- | --- | --- | --- | --- | --- | --- | --- | --- |
| **Response** | Q1 | Q2 | Q3 | Q4 | Q5 | Q6 | Q7 | Q8 | Q9 | Q10 |
| **1 – no difficulty**  **(DQ10 not at all)** | 1604 (99.9) | 1588 (98.9) | 1591 (99.1) | 1535 (95.6) | 1565 (97.4) | 1593 (99.2) | 1553 (96.7) | 1511 (94.1) | 1450 (90.3) | 693 (43.2) |
| **2 – some difficulty**  **(DQ10 same or less)** | 2  (0.1) | 18  (1.1) | 5  (0.3) | 61 (3.8) | 37  (2.3) | 10  (0.6) | 49 (3.1) | 87 (5.4) | 108 (7.7) | 341 (21.2) |
| **3 – a lot of difficulty**  **(DQ10 more)** | 0  (0.0) | 0  (0.0) | 1  (0.1) | 8  (0.5) | 3  (0.2) | 2  (0.1) | 4  (0.2) | 5  (0.3) | 47 (2.9) | 359 (22.4) |
| **4 – cannot do at all**  **(DQ10 a lot more)** | 0  (0.0) | 0  (0.0) | 9  (0.6) | 2  (0.1) | 1  (0.1) | 1  (0.1) | 0  (0.0) | 3  (0.2) | 1  (0.1) | 213 (13.3) |

Ten questions of the WGCFM version used in this study:

1. Does (name) wear glasses of contact lenses?
   1. If child wears glasses: does (name) have difficulty seeing, when wearing his/her glasses?
   2. If child does not wear glasses: does (name) have difficulty seeing?
2. Does (name) use a hearing aid?
   1. If child uses a hearing aid: does (name) have difficulty hearing, when using his/her hearing aid(s)?
   2. If child does not use a hearing aid: Dose (name) have difficulty hearing?
3. Compared with children of the same age, does (name) have difficulty walking?
4. Compared with children of the same age, does (name) have difficulty learning to do new things?
5. Compared with children of the same age, does (name) have difficulty playing with other children?
6. Does (name) have difficulty understanding you?
7. Do you have difficulty understanding what your child wants?
8. Compared with children of the same age, does (name) have difficulty learning the names of common objects?
9. Compared with children of the same age, does (name) have difficulty playing with toys or household objects?
10. Compared with children of the same age how much does (name) kick, bite or hit other children of adults? (either provoked or unprovoked)
