# Supplementary material for: Performance of the UNICEF/UN Washington Group tool for identifying functional difficulty in rural Zimbabwean children
Source: PLoS One. 2022 Sep 16;17(9):e0274664. doi: 10.1371/journal.pone.0274664 (PMC9480986; doi:10.1371/journal.pone.0274664)
Supplement: S1 Fig — (DOCX) [file pone.0274664.s005.docx]

**Supplementary Fig 1. Bar chart of functional difficulty prevalence by cut off in this study and field testing.**

Four values for functional difficulty are provided per study based on the four cut-offs explore in the tool’s field testing. Cut-off 2A is the cut-off recommended by the Washington Group for the 2-4-year-old version of the tool. The penultimate four-bar set show data from this study with the ‘controlling behaviour’ domain included; the final four-bar set show data from this study with the ‘controlling behaviour’ domain excluded). The final four bars (Zimbabwe Excluding Q10) report the disability statistics of this study (removal of the controlling behaviour domain in this study meant no difference between cut-offs 2 and 2A).
