# Supplementary material for: Performance of the UNICEF/UN Washington Group tool for identifying functional difficulty in rural Zimbabwean children
Source: PLoS One. 2022 Sep 16;17(9):e0274664. doi: 10.1371/journal.pone.0274664 (PMC9480986; doi:10.1371/journal.pone.0274664)
Supplement: Attachment — Submitted filename: Comments to the Author_ performance.docx [file pone.0274664.s008.docx]

**Comments to the Authors**

- **Abstract**
- Abstract section needs some editorial corrections as indicated in the track changes
- The structure of the abstract should follow Plose one’s format
- Indicate the design of the study clearly as indicated in the body of the manuscript
- Mention briefly all statistical analyses used
- **Methods:**
- Manuscript should stick to Plose one’s format
- Explain how pregnancy was confirmed
- “The ECD assessment took 2-3 hours and was conducted in the child’s home”. How the investigators overcome resistance that may arise from the respondent’s side?
- Nothing mentioned in the methods section how nutritional indices and dietary diversity were measured unless it was described in other papers. Explain
- **Results**
- Are well organized.
- However, make clear whether the odds ratio mentioned in table 4 is the adjusted one or not.
- Lines 356-370 should appear before Table 5
- **Discussion**
- “Disability was defined by the WGCFM, with concurrent validity assessed using the MDAT as a comparator”. Do we need definition of concept in discussion section?
- The authors should paraphrase the key messages in the first paragraph of the discussion as per the specific objectives of the study before commencing any discussion while highlighting on objectives of the study.
- Summarize discussion of each key finding in a single paragraph
- Implication of each key finding was not discussed in detailbeyond theoretical discussion
- The limitation of the study needs to be described in detail.
- **Conclusion and recommendations should be bold enough**
