# Supplementary material for: Performance of the UNICEF/UN Washington Group tool for identifying functional difficulty in rural Zimbabwean children
Source: PLoS One. 2022 Sep 16;17(9):e0274664. doi: 10.1371/journal.pone.0274664 (PMC9480986; doi:10.1371/journal.pone.0274664)
Supplement: Attachment — Submitted filename: Response to Reviewers.docx [file pone.0274664.s009.docx]

**PONE-D-21-31744**

Performance of the UNICEF/UN Washington Group tool for identifying functional difficulty in rural Zimbabwean children

**PLOS ONE**

Dear PLOS ONE and reviewers,

Many thanks for taking the time to review our article, and for your comments and suggestions. I appreciate that it has been a busy time and that a lot of work has come from you side in getting to this point so again, thank you.

It was gratifying to see that the article was overall well received. We have taken the time to go through each comment in turn: below please find each of your original comments (black) followed by our response (red). Line references in this letter refer to the ‘**Manuscript**’ document.

We very much look forward to your response.

All the best

Tom

Dr Thomas Dunne, Corresponding Author

1. Please ensure that your manuscript meets PLOS ONE's style requirements, including those for file naming. The PLOS ONE style templates can be found at 
<https://journals.plos.org/plosone/s/file?id=wjVg/PLOSOne_formatting_sample_main_body.pdf> and 
<https://journals.plos.org/plosone/s/file?id=ba62/PLOSOne_formatting_sample_title_authors_affiliations.pdf>
These links have been reviewed and the article amended as applicable, many thanks.

2. You indicated that you had ethical approval for your study. In your Methods section, please ensure you have also stated whether you obtained consent from parents or guardians of the minors included in the study or whether the research ethics committee or IRB specifically waived the need for their consent. Consent for participation was obtained from parents or guardians of the children that took part in the study; this is now in text, lines 236-238.

3. Please include a complete copy of PLOS’ questionnaire on inclusivity in global research in your revised manuscript. Our policy for research in this area aims to improve transparency in the reporting of research performed outside of researchers’ own country or community. The policy applies to researchers who have travelled to a different country to conduct research, research with Indigenous populations or their lands, and research on cultural artefacts. The questionnaire can also be requested at the journal’s discretion for any other submissions, even if these conditions are not met.  Please find more information on the policy and a link to download a blank copy of the questionnaire here: <https://journals.plos.org/plosone/s/best-practices-in-research-reporting>. Please upload a completed version of your questionnaire as Supporting Information when you resubmit your manuscript. Please find included in supporting information.

4. We note that you have indicated that data from this study are available upon request. PLOS only allows data to be available upon request if there are legal or ethical restrictions on sharing data publicly. For more information on unacceptable data access restrictions, please see <http://journals.plos.org/plosone/s/data-availability#loc-unacceptable-data-access-restrictions>. ECD data are available on ClinEpi, URL to be provided when we go live (we are currently going through quality control/assurances before it will be live to everyone).

In your revised cover letter, please address the following prompts:

a) If there are ethical or legal restrictions on sharing a de-identified data set, please explain them in detail (e.g., data contain potentially sensitive information, data are owned by a third-party organization, etc.) and who has imposed them (e.g., an ethics committee). Please also provide contact information for a data access committee, ethics committee, or other institutional body to which data requests may be sent. ECD data are available on ClinEpi, URL to be provided when we go live (we are currently going through quality control/assurances before it will be live to everyone).

b) If there are no restrictions, please upload the minimal anonymized data set necessary to replicate your study findings as either Supporting Information files or to a stable, public repository and provide us with the relevant URLs, DOIs, or accession numbers. For a list of acceptable repositories, please see <http://journals.plos.org/plosone/s/data-availability#loc-recommended-repositories>. ECD data are available on ClinEpi, URL to be provided when we go live (we are currently going through quality control/assurances before it will be live to everyone).

We will update your Data Availability statement on your behalf to reflect the information you provide.

5. Please review your reference list to ensure that it is complete and correct. If you have cited papers that have been retracted, please include the rationale for doing so in the manuscript text, or remove these references and replace them with relevant current references. Any changes to the reference list should be mentioned in the rebuttal letter that accompanies your revised manuscript. If you need to cite a retracted article, indicate the article’s retracted status in the References list and also include a citation and full reference for the retraction notice. On re-reviewing cited papers, we could not find any that had been retracted. The reference list has been reviewed to ensure formatting is correct. 1 new reference has been included as per changes in the paper’s discussion section; this is reference 37.

5. Review Comments to the Author

Please use the space provided to explain your answers to the questions above. You may also include additional comments for the author, including concerns about dual publication, research ethics, or publication ethics. (Please upload your review as an attachment if it exceeds 20,000 characters)

Reviewer #1: The article is interesting and well-written. Moreover, the statistical analysis is well-conducted. I have only the following comment.
1. The statistical methods considered by the authors, with a special focus on the regression approaches, rely on assumptions about the nature of the underlying data. If the data do not meet those assumptions, then the results often are not valid. Therefore, it is important for authors to check if those assumptions are satisfied for the data at hand, at least, approximately. Many thanks for kindly reviewing our article and for your comment. In response to this, prior to performing the statistical analysis, all data were assessed for normality to ensure the correct choice of parametric or non-parametric test was made. The method itself (reference 29) has been used in similar studies and was carefully selected; the details of the statistical analysis are found in the article text, from line 186.

Reviewer #2: Comments to the Authors
Many thanks for kindly reviewing our article and for your comments. Please find our responses set out below alongside your original suggestions.

◊ Abstract
• Abstract section needs some editorial corrections as indicated in the track changes PLOS ONE style templates re-reviewed and amendments made, many thanks.

• The structure of the abstract should follow Plos one’s format PLOS ONE Style templates re-reviewed and amendments made, many thanks.

• Indicate the design of the study clearly as indicated in the body of the manuscript. This has now been updated; see ‘Abstract’, lines 31-32.

• Mention briefly all statistical analyses used Updated: see ‘Abstract’, ‘Methods’ (lines 30-40).


◊ Methods:
• Manuscript should stick to Plos one’s format PLOS ONE Style templates re-reviewed and amendments made throughout.

• Explain how pregnancy was confirmed Pregnancy tests were undertaken by village health workers for any women who reported missing a menstrual period; for women who were confirmed pregnant and interested in the trial, a research nurse visited the home and repeated the urinary pregnancy test and also enquired about any recent vaginal bleeding. Any women with vaginal bleeding in the last 2 weeks were not enrolled; they were referred to clinics and re-visited 2 weeks later to ascertain whether they were still pregnant. This has now been inputted to the article text, lines 122-127.

• “The ECD assessment took 2-3 hours and was conducted in the child’s home”. How the investigators overcome resistance that may arise from the respondent’s side? During the field work, measures were put in place to make responding to the surveys as easy and convenient as possible for the consented participants. Specifically, tests were done in the participant’s home at a time convenient for them. Furthermore, breaks were given for as long as required if the children needed time to rest during data collection or if the family had other commitments. This has now been inputted to the article text, lines 178-180.

• Nothing mentioned in the methods section how nutritional indices and dietary diversity were measured unless it was described in other papers. The original study publication includes the information: Assessments included whether an infant was still breast feeding, whether a mother reported the correct ways to feed a child before and after illness, the infant meeting a minimum dietary diversity score in the past 24 hours, and whether in the past 24 hours the infant had consumed food rich in iron, food from an animal, food rich in vitamin a or food including nutributter. The reference for this paper, reference 26, has been added to this section of methods, line 130-131.


◊ Results
• However, make clear whether the odds ratio mentioned in table 4 is the adjusted one or not. The reported odds ratios in table 4 are unadjusted, with odds ratios adjusted in the main regression model. This is now in text, line 318.

• Lines 356-370 should appear before Table 5. This has been addressed.


◊ Discussion
• “Disability was defined by the WGCFM, with concurrent validity assessed using the MDAT as a comparator”. Do we need definition of concept in discussion section? This point has been commented on in the limitations section of the discussion, on from line 473.

• The authors should paraphrase the key messages in the first paragraph of the discussion as per the specific objectives of the study before commencing any discussion while highlighting on objectives of the study. This has been actioned with the first paragraph of the discussion amended. This paragraph now summarises key findings as per the pre-set analysis plan in the methods section. The order or points set out in this paragraph is reflected in the order of paragraphs for the remainder of the discussion section of the article.

• Summarize discussion of each key finding in a single paragraph. The discussion has been reformatted around the key findings. The paragraphs in the discussion section are now:
1. Summary and key points

2. Functional difficulty by WGCGM

3. Severe functional difficulty by WGCFM

4. Mat HIV status and disability

5. WGCFM and MDAT concurrent validity

6. Risk factors for functional difficulty

7. Limitations
8. Summary / conclusion

• Implication of each key finding was not discussed in detail beyond theoretical discussion the final paragraph of the discussion (from line 488) moves on from theoretical discussion to highlight some key applications of this work. Most importantly, the application of the WGCFM in disability assessments in novel population to identify children with functional / severe functional difficulty to allow interventions to aid their functioning and societal integration. Furthermore, with regards to practical application, this paragraph goes on to provide several key starting points for ongoing research based on the findings of this study e.g., exploring ‘problem’ questions in the tool in novel populations, assessing risk factors for functional difficulty / disability in different populations, assessing impact of HIV-exposure in mothers to ECD in their children in other populations.

• The limitation of the study needs to be described in detail. The authors have reflected on the work and from this further developed the limitations section of the article, see strengths and weaknesses paragraph from line 569.


◊ Conclusion and recommendations should be bold enough We have now revised the discussion with regards to layout, using the first paragraph to summarise key findings as per the original plan described the methods section, and further used the order of this paragraph to format the ongoing structure of the discussion. We feel that this gives a clearer structure to the discussion, allowing initial delivery of findings followed by additional discussion around specific key findings. We are appreciative of the comments made around structure of the discussion and now feel it to be bolder, with better clarity.
